# A real-time, quantitative PCR method using hydrolysis probes for the monitoring of *Plasmodium falciparum *load in experimentally infected human volunteers

**DOI:** 10.1186/1475-2875-10-48

**Published:** 2011-02-28

**Authors:** Rebecca J Rockett, Sarah J Tozer, Chris Peatey, Seweryn Bialasiewicz, David M Whiley, Michael D Nissen, Katharine Trenholme, James S Mc Carthy, Theo P Sloots

**Affiliations:** 1 Queensland Paediatric Infectious Diseases Laboratory, Queensland Children's Medical Research Institute, Queensland Children's Health Service and The University of Queensland, Queensland, Australia; 2 Microbiology Division, Pathology Queensland Central, Royal Brisbane and Women's Hospital Campus, Queensland, Australia; 3 Malaria Biology Laboratory, Infectious and Tropical Diseases, Queensland Institute of Medical Research, Queensland, Australia; 4 Clinical Tropical Medicine Laboratory, Queensland Institute of Medical Research, University of Queensland, Queensland, Australia

## Abstract

**Background:**

The accurate quantification of *Plasmodium falciparum *parasite numbers by PCR is an important tool for monitoring growth kinetics in subjects infected and subsequently treated with anti-malarial agents.

**Methods:**

A real-time quantitative PCR (rt-qPCR) method using primers and a hydrolysis probe that targets the 18S rRNA gene was adapted and optimized to estimate parasite load in blood samples. Samples included laboratory prepared blood samples of varying parasite concentrations (6.4 × 10^5 ^to 6.4 parasites per 500 μl of packed red blood cells (500pRBC)) and blood samples collected from an experimentally infected human subject collected at 19 time points over 10 days. Sample preparation and extraction, detection chemistry, assay reproducibility, and limit of detection were compared to a previously published SYBR Green rt-qPCR used in a malaria vaccine clinical trial.

**Results:**

Both the rt-qPCR hydrolysis probe assay and SYBR Green rt-qPCR provided a limit of detection of 6.4 × 10^1 ^parasites per 500pRBC. However non-specific amplification in the SYBR Green rt-qPCR assay led to either inaccurate estimation of parasite load at levels below 6.4 × 10^2 ^parasites per 500pRBC and to false-positive detection of parasites in negative samples. The rt-qPCR hydrolysis probe assay was specific and provided reliable quantification of parasitaemia down to 6.4 × 10^1 ^parasites per 500pRBC. Notably, 12 of the 19 consecutive samples collected from the experimentally infected subject were at or below 6.4 × 10^2 ^copies per 500pRBC.

**Conclusions:**

These results show that the hydrolysis probe rt-qPCR assay is superior to the SYBR Green rt-qPCR for the quantification of *P. falciparum *in human blood samples. The hydrolysis probe rt-qPCR is now in use in the Queensland paediatric infectious diseases laboratory (QPID) to monitor parasitaemia in experimentally-infected clinical trial subjects.

## Background

Approximately one half of mankind is at risk of malaria infection, with an estimated 243 million cases occurring in 2008, resulting in an estimated 863,000 million deaths [1]. The development of effective and affordable anti-malarial drugs and vaccines is crucial in the fight against malaria. To assess the efficacy of such anti-malarial agents in clinical trials, sensitive and reliable laboratory detection methods are required.

Until recently, the gold standard method for the diagnosis of malaria was examination of thin and thick blood films, which has a predicted limit of detection of 5 to 20 parasites per microlitre of blood [2]. Subsequently, a range of diagnostic PCR assays have been developed and these increase the sensitivity of detection of blood-stage malarial parasites by at least a hundred-fold compared with traditional microscopy [[3,4], and [5]]. Recently real-time quantitative PCR (rt-qPCR) assays have been specifically developed for use in malaria vaccine clinical trials, successfully demonstrating quantitative changes in parasitaemia [6]. These methods included a filtration step to remove leukocytes, and hence human DNA, from the blood during sample preparation, and, have used the intercalating dye SYBR Green for the detection of amplification products.

Previously, the QPID laboratory described an rt-PCR method for the sensitive detection and differentiation of four Plasmodium species, including *Plasmodium falciparum*, using a common primer set and species-specific hydrolysis probes targeting the 18S rRNA gene [7]. This paper describes the adaptation of this method to allow quantitative monitoring of changes in parasitaemia in human subjects, and compared it to the SYBR Green rt-qPCR method described by Andrews and colleagues [6].

The development of a hydrolysis probe rt-qPCR method, sought to establish that the assay performed with at least equal sensitivity and specificity to the SYBR Green rt-qPCR method described previously by Andrews *et al *[6]. This was achieved by determining the experimental limit of detection of the two assays, and by assessing their performance in the monitoring of parasitaemia in the blood of an experimentally infected subject. In addition, with the use of a specific hydrolysis probe in the assay design it was determined if there is a need to remove leukocytes from the patient's blood.

## Methods

### Control specimens prepared for the evaluation of the rt-PCR assays

Control standards were prepared for the comparative evaluation of the SYBR Green rt-qPCR and hydrolysis probe rt-qPCR methods. Specifically, malaria parasite cultures of the *P. falciparum *3D7 strain were maintained as described previously [8], and were grown to between 3-7% parasitaemia. Two to four millilitres of this culture were prepared for fluorescence-activated cell sorting (FACS) as previously described [9]. Ethidium bromide (EtBr) stained cultures were sorted by a FACS Aria live cell sorter (Becton Dickson, San Jose, CA) into 96 well plates. A known number of parasites were sorted into specific wells, ranging from 10 to 10^4 ^parasites. These parasites were able to be re-stained with EtBr at a later time point and counted using a FACS Canto II (Becton Dickson, San Jose, CA) with a high throughput sampler option (HTS), to verify the number of parasites present.

The control standards were prepared from the *P. falciparum *culture described above, as follows; laboratory-infected blood samples were diluted in parasite-negative whole blood or water to give final parasite concentrations of 6.4 × 10^5^, 6.4 × 10^4^, 6.4 × 10^3^, 6.4 × 10^2^, 6.4 × 10^1 ^parasites per 500pRBC. Standards of high parasite load (6.4 × 10^5^, 6.4 × 10^4 ^and 6.4 × 10^3 ^parasites per 500pRBC) were tested in triplicate; lower standards (6.4 × 10^2^, 6.4 × 10^1^, and 6.4 parasites per 500pRBC) were tested in replicates of ten.

### Specimens from an experimentally infected volunteer

Nineteen sequentially collected blood samples from a human volunteer who was experimentally infected with *P. falciparum *3D7 were used for the validation of the quantitative PCR assays. This volunteer was a participant in a larger study of the *in vivo *efficacy of anti-malarial drug, to be described in more detail elsewhere [McCarthy *et al*, manuscript in preparation]. Briefly, a blood sample was taken pre-infection and then every 12 hours from Day 3 at 8 am, for 10 consecutive days. Anti-malarial treatment was administered on Day 7 at 8 pm, and two extra blood samples were collected post treatment at +6 and +18 hours). Following extraction of the parasite DNA in these samples, extracts were tested in triplicate to validate the rt-qPCR assays. Thick and thin blood film preparations were also used to monitor the volunteer's parasitaemia at each time point.

### Ethics approvals

The collection and analysis of samples described in this study received the requisite approval from the Royal Brisbane and Women's Hospital Human Research Ethics Committee (approval number 2008/003) and the Queensland Institute for Medical Research Human Ethics Committee (approval number p1286), respectively. The clinical trial from which the patient samples were collected was registered at ClinicalTrials.gov (NCT01055002).

### Sample preparation and nucleic acid extraction

FACs counted *P. falciparum *control standards (described above) were used to determine the need for leukocyte removal by filtration as described by Andrews *et al *[6]. It was assumed that the removal of unrelated (human) DNA would improve the specificity of a SYBR Green rt-qPCR assay, but is not necessary for the more specific hydrolysis probe rt-qPCR. Fresh control standards were diluted with parasite free whole blood to final parasite concentrations of 5.0 × 10^5^, 1.0 × 10^5^, 2.0 × 10^3^, 1.0 × 10^3^, 5.0 × 10^2^, 2.5 × 10^2^, 1.0 × 10^2 ^parasites per 500pRBC. These sample dilutions were then processed with and without Plasmodir filtration. Briefly, samples for filtration were centrifuged and plasma was removed. To each sample 2.5 ml of sterile PBS was added, the suspension was mixed by inversion, and passed through a Plasmodipur filter (Euro Diagnostica BV, Arnhem, The Netherlands). An additional 3.5 ml of sterile PBS was used to flush the filter and wash out remaining red blood cells (RBC). Samples were then centrifuged and supernatant was removed. 500 μl of the RBC pellet was taken for immediate DNA extraction, and the remainder stored at -80°C.

For unfiltered samples, 2 ml of whole blood was centrifuged for 5 minutes at 2500 rpm. 500 μl of packed red cells was removed and added to 500 μl of PBS and mixed thoroughly. The packed red blood samples were split into two 1.5 ml tubes containing 500 μl in each aliquot. The DNA from one of these was extracted immediately, and the second aliquot was stored at -80°C.

Before DNA extraction, 10^4 ^copies of equine herpes virus (EHV) DNA (Cp = 30) were added to each of the filtered and unfiltered sample dilutions to monitor the efficiency and reproducibility of the extraction process [10]. Nucleic acid was then extracted using the QIAamp DNA Mini kit (QIAGEN, Australia) following the manufacturer's instructions. Extraction of nucleic acid was achieved by adding 40 μl of Qiagen Protease K and incubating each sample at 56°C for 10 minutes. 400 μl of 100% ethanol was added and mixed thoroughly. Nucleic acid extracts were eluted in 100 μl of elution buffer and stored at -80°C until PCR reactions were performed. After extraction each standard was tested by PCR in triplicate, and quantified using a standard curve of predetermined parasitaemia.

### Real-time quantitative PCR methods

#### SYBR green rt-qPCR assay

This method was adapted from the assay previously described by Andrews *et al *[6] Briefly, the PCR mix consisted of 12.5 μl Quantitect SYBR Green PCR Mix (Qiagen, Australia), 0.3 μM of each primer (18SRank1F: GTT CTG GGG CGA CTA T and 18SRank1R: TGC ATC ACC ATC CAA G), 4 mM of MgCl_2 _and 5 μl of template DNA in a 25 μl final reaction mix. PCR was performed in a Rotorgene 3000 or 6000 (Qiagen, Australia) under the following conditions: 15 minutes incubation at 95°C, followed by 45 cycles of 94°C for 5 seconds, 50°C for 15 seconds and 72°C for 10 seconds. A melt curve analysis step was added with a melting profile of 50 - 94°C at 1°C per second.

#### Hydrolysis probe rt-qPCR assay

The oligonucleotides and cycling conditions used were those previously developed in the QPID laboratory [7]. Briefly, the reaction mix consisted of 12.5 μl Quantitect Probe PCR Mix (Qiagen, Australia), 10 pmol of each primer (PerFAL-Forward: CTT TTG AGA GGT TTT GTT ACT TTG AGT AA and PerFAL-Reverse: TAT TCC ATG CTG TAG TAT TCA AAC ACA), 4 pmol of probe (PerFAL-probe: Fam-TGT TCA TAA CAG ACG GGT AGT CAT GAT TGA GTT CA-BHQ1) and 5 μl of template DNA in a 25 μl final reaction volume. Amplification was performed in a Rotorgene 3000 or 6000 (Qiagen, Australia) under the following conditions: 15 minutes incubation at 95°C, followed by 45 cycles of 95°C for 15 seconds and 60°C for 1 minute.

#### Standard curve

Serial dilutions were prepared from laboratory cultures of *P. falciparum*. Six standards were used on each run ranging from 6.4 × 10^5 ^to 6.4 parasites per 500pRBC and two negative controls consisting of sterile water. Parasite concentration was extrapolated from crossing point (Cp) values by a simple linear regression model in the form y = mx+b using Qiagen, Rotorgene software (Qiagen, Australia).

## Results

### Sensitivity and specificity of rt-qPCR assays

Serial dilutions of FACs counted *P. falciparum *3D7 infected RBC were used to evaluate the sensitivity and specificity of each assay. The limit of detection for both the rt-qPCR hydrolysis probe assay and SYBR Green rt-qPCR is, 6.4 × 10^1 ^parasites per 500pRBC (Table 1). Interestingly, amplification of parasite DNA was affected by the medium used to dilute the infected RBC. Cp values were reduced by approximately two cycles with the addition of parasite-negative blood nucleic acid extract using the rt-qPCR SYBR Green assay but not in the hydrolysis probe rt-qPCR. This suggests that the SYBR Green assay may non-specifically amplify human DNA and thereby affects the accuracy of parasite quantification. The reliable detection of 6.4 parasites per 500pRBC was limited in both assays with only one replicate being amplified in both water and parasite-negative blood extract dilutions using the hydrolysis probe assay. However, the SYBR Green rt-qPCR assay showed amplification in all 10 replicates at the 6.4 parasites per p500pRBC dilution in parasite-negative whole blood extract, and only one positive replicate in extracts diluted in water. Melt analysis revealed that 7 of 10 amplification products showed a shift in melt peak from 82°C a temperature which is characteristic of a specific amplification product to 79°C, which is indicative of unrelated amplicons. Non-specific melt peaks were also noted in seven out of 10 amplification reactions at 64 parasites per 500pRBC diluted in parasite-negative blood extract.

**Table 1:** Sensitivity and specificity of rt-qPCR Assays using hydrolysis probes and SYBR Green to quantify laboratory prepared dilutions of *Plasmodium falciparum*

Dilutions (replicates)		Hydrolysis probe	SYBR Green
		*Crossing point (SD), Quant value (SD)*	

**In Water**	6.4 × 10^5 ^(×3)	24.6 (0.06), 670 000 (28 000)	20.5 (0.10), 740 000 (48 000)
	6.4 × 10^4 ^(×3)	28.2 (0.09), 60 000 (3 700)	24.6 (0.68), 57 000 (27 000)
	6.4 × 10^3 ^(×3)	30.8 (0.50), 9 200 (1 600)	27.6 (0.38), 8 100 (1 600)
	6.4 × 10^2 ^(×10)	34.6 (0.40), 550 (140)	32.9 (0.57), 550 (200)
	6.4 × 10^1 ^(×10)	37.9 (0.55), 56 (20)	36.1 (1.46), 90 (70)
	6.4 (×10)	37.9 (n/a), 52 (n/a)	40.39 (n/a), 4 (n/a)

**In Negative Extract**	6.4 × 10^5 ^(×3)	24.7 (0.02), 650 000 (8 600)	23.0 (0.22), 710000 (100 000)
	6.4 × 10^4 ^(×3)	28.1(0.20), 62 000 (8 400)	26.6 (0.21), 61 000 (8 200)
	6.4 × 10^3 ^(×3)	30.7 (0.41), 6 800 (1 200)	30.1 (0.44), 7,600 (2 600)
	6.4 × 10^2 ^(×10)	33.5 (0.24), 660 (120)	34.7 (0.37), 410 (120)
	6.4 × 10^1 ^(×10)	37.2 (1.09), 60 (50)	37.2^a ^(1.22), 90 (90)
	6.4 (×10)	37.6 (n/a), 40 (n/a)	39.7^a ^(1.75), 25 (45)

^a ^Indicates nonspecific melting peaks of 79°C obtained by melting curve analysis of replicates tested.

n/a = Not Applicable

SD = Standard Deviation

### Removal of leukocytes from blood samples

Pre-treatment of the *P. falciparum *control standards by Plasmodir filtration showed that there was no significant difference in both the Cp values and the estimated number of parasites for each dilution tested. These results were consistent when tested by either the SYBR Green rt-qPCR assay or the hydrolysis probe rt-qPCR assay (Table 2). Interestingly, filtration did not remove non specific amplification using the SYBR Green assay, with melting peaks of 79°C seen at < 5.0 × 10^2 ^in both filtered and unfiltered standards.

**Table 2:** Evaluation of Filtration methods to improve sensitivity and specificity of rt-qPCR assays

Dilutions (*3 × replicates*)		Hydrolysis probe assay		SYBR Green assay	
		*Crossing point (SD), Quant value (SD)*			

**Filtered**	5.0 × 10^5^	23.38 (0.03),	540 000 (9 000)	20.95 (0.68)	850 000 (400 000)
	1.0 × 10^5^	25.36 (0.16)	160 000 (16 000)	24.20 (0.44)	81 000 (26 000)
	2.0 × 10^4^	28.33 (0.17)	27 000 (3000)	26.14 (0.31)	20 000 (4000)
	1.0 × 10^3^	33.74 (0.44)	1000 (300)	32.65 (0.49)	200 (70)
	5.0 × 10^2^	34.64 (0.45)	600 (160)	33.29 (1.24)	160 (130)
	2.5 × 10^2^	37.40 (n/a)	110 (n/a)	37.02 (1.05)^a^	10 (10)
	1.0 ×02	Not detected		False Positive	20 (10)

**Unfiltered**	5.0 × 10^5^	24.53 (0.62)	300 000 (94 000)	22.83 (0.46)	200 000 (70 000)
	1.0 × 10^5^	25.46 (0.35)	156 000 (33 000)	24.50 (0.11)	64 000 (4800)
	2.0 × 10^4^	29.15 (0.05)	17 000 (500)	29.85 (0.44)	1500 (500)
	1.0 × 10^3^	33.81 (0.38)	1000 (220)	32.98 (0.16)	160 (20)
	5.0 × 10^2^	35.27 (1.63)	550 (450)	36.38 (0.23)^a^	15 (3)
	2.5 × 10^2^	38.35 (1.06)	75 (50)	35.63 (0.73)^a^	30 (10)
	1.0 ×02	Not detected		False Positive	10 (2)

^a ^Indicates nonspecific melting peaks of 79°C obtained by melting curve analysis of replicates tested.

n/a = Not Applicable

SD = Standard Deviation

ND = Not Detected

### Validation of the rt-qPCR methods

Both the SYBR Green and hydrolysis probe rt-qPCR were evaluated for their ability to accurately monitor parasitaemia in an experimentally infected subject (Table 3). The hydrolysis probe rt-qPCR did not detect parasite DNA in any of the triplicate extracts from the pre-infection control, whereas the SYBR Green rt-qPCR recorded a positive result for each of the three replicates (Table 3). Similarly, no parasites were detected in the samples collected at Day 4 pm and Day 10 pm by the hydrolysis probe rt-qPCR, yet showed two of three positive results and one of three positive results with the SYBR Green assay respectively. Further characterization of these amplification products by melting peak analysis showed that the product amplified with the SYBR Green assay gave a melting temperature of 79°C which was characteristic of non-specific amplification, compared to the expected melting temperature of 82°C for the specific amplicon. Apart from the above, both assays showed positive results in at least one replicate of samples collected after three days following inoculation. The SYBR Green rt-qPCR showed non-specific amplification in all replicates at 3, 4, 6, 9 and 10 days post infection (melting temperature of 79°). No parasites were detected in any of the blood films (thin and thick) collected from this subject during the course of the trial.

**Table 3:** Quantification of parasitaemia in a experimental infected subject, over 10 days and 19 time points with two rt-qPCR assays

	Hydrolysis probe assay			SYBR Green assay			
**Dilutions (*3 × replicates*)**	***Replicates positive***	***Cp,(SD)***	***Quant value (SD)***	***Replicates positive***	***Cp,(SD)***	***Quant value (SD)***	***Specific melt 82°C***

Pre-infection	0/3	ND		3/3	36.8 (0.13)^a^	False Positive	0/3
Day 3 AM	1/3	39.6 (n/a)	18 (n/a)	3/3	37.4 (0.29)^a^	10 (3)	2/3
Day 3 PM	2/3	38.1 (1.2)	60 (40)	3/3	36.8 (0.44)^a^	20 (7)	0/3
Day 4 AM	3/3	38.4 (0.59)	40 (20)	3/3	36.8 (0.83)^a^	20 (15)	1/3
Day 4 PM	0/3	ND		3/3	36.3 (0.23)^a^	False Positive	2/3
Day 5 AM	3/3	33.9 (0.27)	900 (180)	3/3	33.2 (0.45)	320 (100)	3/3
Day 5 PM	3/3	33.2 (0.04)	1400 (40)	3/3	32.3 (0.64)	700 (300)	3/3
Day 6 AM	3/3	37.7 (0.85)	70 (40)	3/3	36.9 (0.80)^a^	20 (13)	2/3
Day 6 PM	3/3	30.3 (0.14)	10100 (1000)	3/3	29.6 (0.68)	5900 (3000)	3/3
Day 7 AM	3/3	29.2 (0.23)	22000 (3300)	3/3	27.3 (0.30)	32000 (7600)	3/3
Day 7 PM *	3/3	31.2 (0.07)	5600 (270)	3/3	29.8 (0.62)	4900 (2100)	3/3
Day 7 PM+6 hrs	3/3	33.5 (0.55)	1200 (400)	3/3	31.7 (0.19)	1100 (160)	3/3
Day 8 AM	3/3	34.8 (0.13)	460 (40)	3/3	33.0 (0.39)	400 (130)	3/3
Day 8 AM+6 hrs	3/3	35.7 (0.91)	300 (140)	3/3	34.4 (0.30)	130 (30)	3/3
Day 8 PM	3/3	37.1 (1.42)	130 (100)	3/3	34.4 (0.45)	140 (40)	3/3
Day 9 AM	2/3	38.9 (0.38)	30 (20)	3/3	35.0 (0.10)^a^	80 (6)	2/3
Day 9 PM	1/3	39.35 (n/a)	20 (n/a)	3/3	36.1 (0.44)^a^	40 (10)	1/3
Day 10 AM	1/3	39.05 (n/a)	30 (n/a)	3/3	37.0 (1.08)^a^	20 (18)	1/3
Day 10 PM	0/3	ND		3/3	36.8 (0.09)^a^	False Positive	1/3

*Administration of anti-malarial treatment at this time point.

^a ^Indicates nonspecific melting peaks of 79°C obtained by melting curve analysis of replicates tested.

Cp = Crossing point

n/a = not applicable

SD = Standard Deviation

ND = Not Detected

## Discussion

Sensitive and reliable malaria rt-qPCR assays are becoming increasingly important for a range of purposes in clinical studies of human malaria. To date SYBR Green rt-qPCR assays have been the most widely used methods for the quantification of parasite load. However, from a technical point of view, the SYBR Green detection method has some limitations. It is an intercalating dye that binds to all double-stranded DNA produced during a PCR reaction, including non-specific amplification products from unrelated nucleic acids present in the sample, such as human DNA extracted from leukocytes. This limitation does not apply for rt-qPCR using hydrolysis probes, as the detection signal is only produced from complementary DNA sequence. Therefore, the use of a hydrolysis probe rt-qPCR results in specific amplification, increasing the accuracy of the estimated parasitaemia in the blood sample. In previous studies, researchers have included a filtration step in the sample preparation procedure, to deplete excess human genomic DNA by removing leukocytes. However, this procedure is cumbersome, time consuming and expensive.

The rt-qPCR method described here does not require the additional filtration step as the more specific hydrolysis probe detection method removes the problem of non-specific amplification. The results obtained in this study showed that the limit of detection of the two assays was similar at 64 parasites per 500pRBC. However, it was demonstrated by melting curve analysis that some of the SYBR Green assay results may be affected by non-specific binding of the intercalating dye, leading to inaccurate estimation of parasitaemia.

This phenomenon was also evident in the validation of the assay using samples collected from an experimentally infected subject, further suggesting that the hydrolysis probe rt-qPCR assay is superior when used for the estimation of parasitaemia in human clinical studies. This was supported by the results which show that the removal of leukocytes from EDTA whole blood by filtration was not necessary, when the hydrolysis probe rt-qPCR assay was used.

In the clinical trial samples, both rt-qPCR assays detected parasites in the majority of blood samples collected during the course of the trial. However, no parasites were detected in any samples by traditional blood film microscopy, reflecting the superior sensitivity of the molecular methods.

It should be noted however, that the rt-qPCR method described can only be applied accurately in the relative quantification of parasitaemia in sequentially collected samples. If this method were to be used for quantification of parasites in individual samples, then minor variation in any of several assay parameters (including extraction efficiency and PCR reproducibility) may lead to significant variation in results and therefore further assay quality assurance should be utilized. This was not necessary for the monitoring of parasitaemia in experimentally infected subjects, because sequentially collected blood samples were tested in a single assay.

## Conclusions

This paper describes a sensitive and specific rt-qPCR protocol using a TaqMan hydrolysis probe to monitor the presence and replication kinetics of *P. falciparum *in experimentally infected human subjects. This method is now in use in the QPID laboratory to monitor parasitaemia before and, after treatment with an anti-malarial agent. Clear improvements were recorded in processing times and specificity when compared to previously published SYBR Green rt-qPCR assay [6]. The hydrolysis probe rt-qPCR assay has subsequently become our method of choice for monitoring anti-malarial drug efficacy in clinical trial subjects.

## Competing interests

The authors declare that they have no competing interests.

## Authors' contributions

RR carried out molecular testing, assay validation and drafted the manuscript. SJT participated in molecular testing. CP carried out the preparation and FACS of control parasites. SB participated in molecular testing. DMW helped to draft the manuscript. MDN participated in assay design and analysis of results. KT was responsible for quantitation and preparation of the infective dose. JSMc developed and managed the *in vivo *efficacy of anti-malarial drug trial and helped to draft the manuscript. TPS participated in assay design and project management and prepared the manuscript for publication. All authors read and approved the final manuscript.
